# A new method for identifying rapid decline dynamics in wild vertebrate populations

**DOI:** 10.1002/ece3.596

**Published:** 2013-06-14

**Authors:** Martina Di Fonzo, Ben Collen, Georgina M Mace

**Affiliations:** 1 Institute of Zoology, Zoological Society of LondonRegent's Park, London, NW1 4RY, UK; 2 Division of Ecology and Evolution, Imperial College LondonSilwood Park, Ascot, SL5 7PY, UK; 3 ARC Centre of Excellence for Environmental Decisions, the NERP Environmental Decisions Hub, Centre for Biodiversity and Conservation Science, University of QueenslandBrisbane, Queensland, 4072, Australia; 4 Department of Genetics, Evolution and Environment, University College LondonDarwin Building, Gower Street, London, WC1E 6BT, UK

**Keywords:** Conservation prioritization, curve fitting, extinction risk, second derivative switch points, threatening process

## Abstract

Tracking trends in the abundance of wildlife populations is a sensitive method for assessing biodiversity change due to the short time-lag between human pressures and corresponding shifts in population trends. This study tests for proposed associations between different types of human pressures and wildlife population abundance decline-curves and introduces a method to distinguish decline trajectories from natural fluctuations in population time-series. First, we simulated typical mammalian population time-series under different human pressure types and intensities and identified significant distinctions in population dynamics. Based on the concavity of the smoothed population trend and the algebraic function which was the closest fit to the data, we determined those differences in decline dynamics that were consistently attributable to each pressure type. We examined the robustness of the attribution of pressure type to population decline dynamics under more realistic conditions by simulating populations under different levels of environmental stochasticity and time-series data quality. Finally, we applied our newly developed method to 124 wildlife population time-series and investigated how those threat types diagnosed by our method compare to the specific threatening processes reported for those populations. We show how wildlife population decline curves can be used to discern between broad categories of pressure or threat types, but do not work for detailed threat attributions. More usefully, we find that differences in population decline curves can reliably identify populations where pressure is increasing over time, even when data quality is poor, and propose this method as a cost-effective technique for prioritizing conservation actions between populations.

## Introduction

One approach to counteracting the world's failure to meet the Convention on Biological Diversity's target of “achieving a significant reduction in the rate of biodiversity loss by 2010” (Convention on Biological Diversity 2002; Butchart et al. 2010) could be achieved through more proactive conservation actions, which tackle potential wildlife losses before it is too late. Studying the impact of anthropogenic activity at the population level is particularly useful as this is also the scale at which pressure first impacts a species; population decline therefore is a prelude to species extinction (Ceballos and Ehrlich 2002; Collen et al. 2009). The status of wildlife populations is also a more sensitive indicator of biodiversity change compared species extinction due to the shorter time-lag between human impact and corresponding shifts in population trends (Ceballos and Ehrlich 2002; Balmford et al. 2003). In addition to understanding the extinction risk of species, identifying changes in wildlife population dynamics can provide information on how populations respond to management to inform future management decisions (Yoccoz et al. 2001).

**Figure 1:**
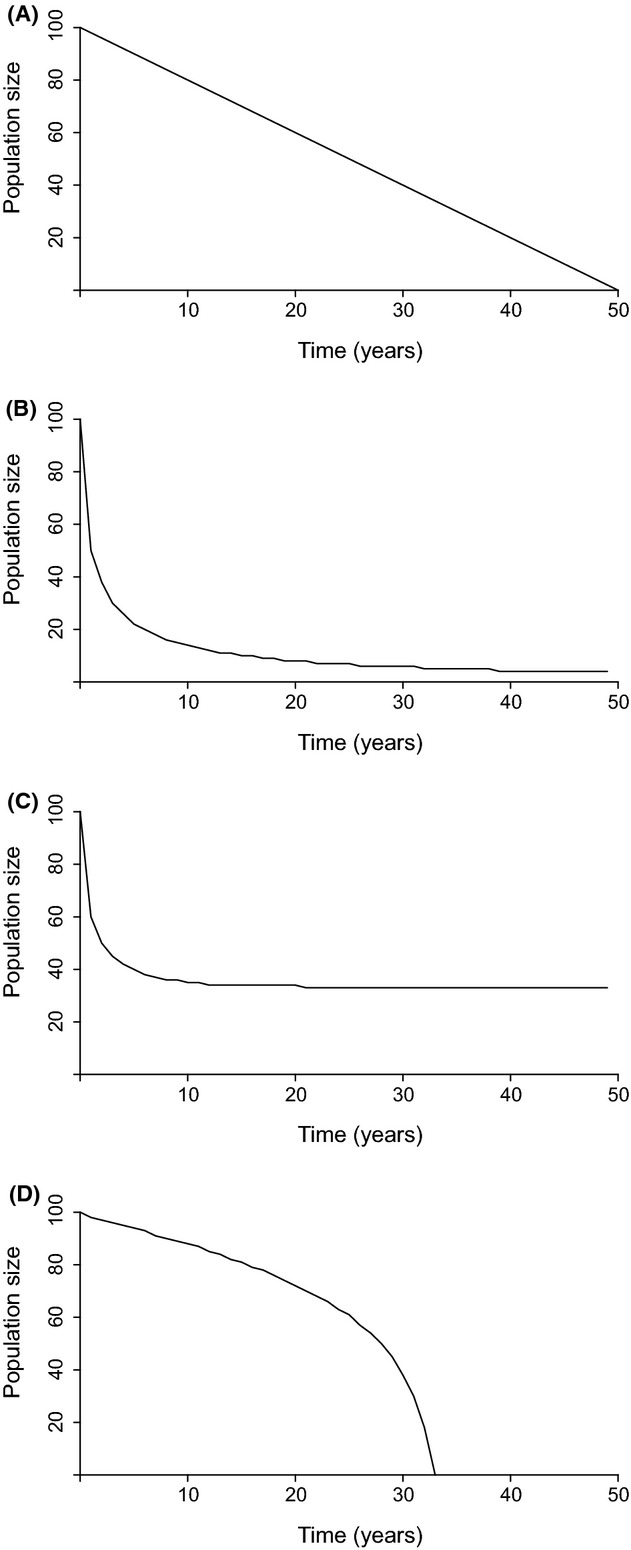
Different types of population decline (reproduced from Mace et al. 2008). Each graph shows population size declining over time in response to (A) a constant removal of individuals, (B) a proportional removal, (C) a proportional removal down to a sustainable level, and (D) an increasing removal of individuals over time.

Although seemingly straightforward, detecting declines can be both under or overestimated by measurement and/or process error (Wilson et al. 2011). Mace et al. (2008) proposed that populations affected by different types of pressure should have different shaped decline curves, depending on the manner in which pressure-induced mortality occurs over time. For instance, a population affected by a constant loss of individuals each year (e.g., under fixed quota harvesting regimes such as the commercial hunting of Kangaroos; Pople and Grigg 1999) should exhibit a linear decline, with an increasing decline rate as the population becomes smaller (Fig. 1A). If a population is affected by a slowing pressure, such as a proportional reduction in harvested individuals over time (e.g., characteristic of the “constant harvest rate strategy” used in fisheries to calculate total allowable catch; Hjerne and Hansson 2001) then it should decline in a concave, exponential manner, with slowing rate of decline as the population reduces in size (Fig. 1B). Such a characteristic decline type may also occur when the number of individuals harvested decreases over time, leading to stabilization at a lower population size, e.g., as a result of the implementation of a managed harvesting program (Fig. 1C). Finally, Mace et al. (2008) proposed that a population that loses an increasing number of individuals over time should decline in a quadratic, convex manner, with an increasing decline rate as the population reduces in size (Fig. 1D). This type of pressure may be caused by “contagious” habitat fragmentation (Boakes et al. 2009) or due to increasing hunting pressure as a result of increasing economic or social value of a species with increasing rarity (Courchamp et al. 2006). A population declining in such a manner could also be experiencing inverse density dependence, which would also cause decline rate to increase as the population reduces in size (Allee 1931; Myers et al. 1995; Courchamp et al. 1999, 2006).

We set out to test this decline-curve approach, both in principle through model simulations and in practice by addressing the following questions using a dataset of wildlife population abundance time-series:

1. How robust is the identification of Mace et al.'s (2008) decline-curve types for different pressure types under varying life history and data quality scenarios?
2. Is it possible to detect differences in decline-curve types in natural populations, relevant to different conservation priorities?
3. How do diagnosable decline-curve types correspond to threatening processes reported for them?

## Methods

This analysis was set in the context of the population information used in calculating vertebrate population trends for the Living Planet Index (LPI), which is a global, composite index tracking overall changes in vertebrate abundance since 1970 (Loh et al. 2005; Collen et al. 2009; McRae et al. 2012). It includes population abundance estimates for about 12,000 time-series, varying from 3 to 100 years in length, across 2500 vertebrate species, including 443 mammals. This allowed us to specify relevant parameter values for key demographic and life-history variables for both the method development and its application.

### Simulation of decline-curve dynamics

We validated the theoretical underpinning of the decline-curve approach by simulating population abundance data for mammal species with slow, medium and fast life-history speeds, under different harvesting regimes to represent the pressure types described in Mace et al. (2008). We chose to simulate a range of life-histories to examine how well the decline-curve patterns could be generalized across species. We used the logistic model of density-dependent growth (eq. 1; Verhulst 1838), with added environmental stochasticity:


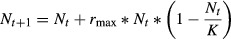
(1)

where *N*_*t+1*_ represents the population size in the next year, *N*_*t*_ the population size in the current year, *r*_max_, the maximum intrinsic rate of population growth, and *K* is the carrying capacity (Verhulst 1838). All analyses were conducted in R.2.12.1. (R Development Core Team 2012). We represented the populations using a simple scalar model to mirror the type of data collected through basic monitoring schemes, in which detailed demographic information is rarely available (Collen et al. 2009). In order to simulate natural fluctuations in abundance, we incorporated environmental stochasticity by sampling *r*_max_ and *K* from random normal distributions, truncated at a lower threshold of 0. Mean parameter values (and corresponding standard deviations) chosen for populations with different life-history speeds are summarized in Table 1. The *r*_max_ values and coefficient of variation (C.V) in *r*_max_ for slow, medium, and fast life-histories are based on estimates validated by species experts and published summaries (Fowler 1988; Gaillard et al. 2000; Jones et al. 2009; E. J. Milner-Gulland pers. comm.). We set C.V in *K* to an arbitrary value of 0.01 across all populations, and the upper thresholds of *N* and *r*_max_ to three standard deviations greater than their respective mean values. We did not include demographic stochasticity in the models as we were not interested in the dynamics of small populations, where this type of stochasticity may mask patterns of external pressure.

**Table 1:** Basic life-history speed parameters used in population simulations

	Model parameter values								
	
Life-history speed	Mean *r*_max_	C.V. of *r*_max_	S.d of *r*_max_	Upper threshold *r*_max_	*N*_1_	*K*	C.V of *K*	SD of *K*	Upper threshold *N*
Slow	0.1	0.15	0.015	0.15	100	1000	0.01	10	1300
Medium	0.2	0.15	0.03	0.3	100	1000	0.01	10	1600
Fast	0.3	0.2	0.06	0.5	100	1000	0.01	10	1900

*r*_max_ represents the intrinsic rate of growth, *K* represents carrying capacity, C.V stands for coefficient of variation, SD for standard deviation, and *N* for population size, with *N*_1_ being the population size at the start of the simulation.

**Figure 2:**
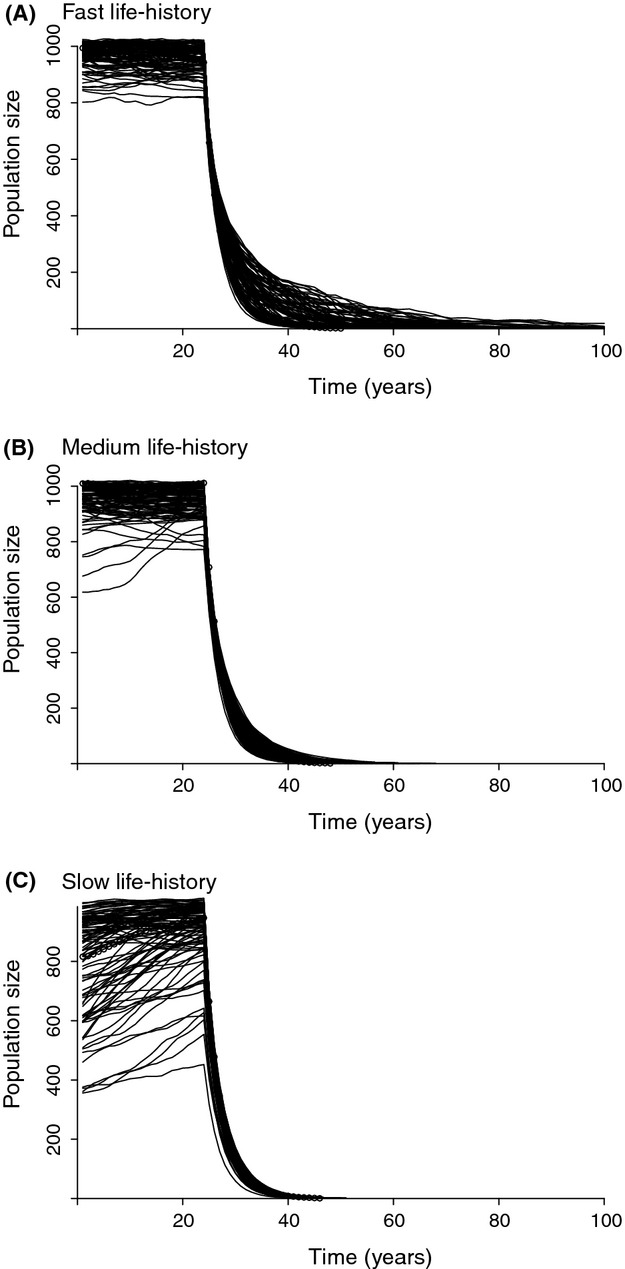
Illustration of 100 simulations of populations with (A) fast, (B) medium, and (C) slow life-history speeds under 30% proportional pressure, with coefficient of variation (C.V) in *K* of 0.01. Pressure was applied from year 25 onwards.

We imposed realistic temporal autocorrelation by specifying the mean of the distributions for parameter values in year t as equal to those in year *t*−1. 10,000 simulations were generated for a period of 150 time-steps for each population model. A population was deemed extinct when its total size fell below one. For each of the 10,000 simulations, the first 50 years of data were discarded in order to allow the population to stabilize, and a random selection of 1000 of the simulations that survived for longer than either 25 or 50 years post stabilization (depending on the simulation scenario imposed) were stored for analysis. Pressure was imposed from year 75 onwards according to different scenarios by removing individuals each year after population renewal through two harvesting strategies: a simulated removal of a fixed number of individuals (*F*; to represent density-independent threats, such as disease or certain overexploitation regimes), or a simulated removal of a proportion of the total population (P; to represent density-dependent threats, such as proportional exploitation regimes or the effects of habitat loss and degradation; Getz and Haight 1989). We imposed a range of intensities for each removal type, summarized in Table 2 (fully described values are in Table S1.). In all cases, pressure was imposed each year on the simulated population until the population went extinct or the simulation period ended. Figure 2 shows an example of 100 simulations of populations with different life-history speeds, affected by 30% proportional pressure.

**Table 2:** Pressure scenarios imposed on populations with slow, medium, and fast life-history speeds

Scenario code	Pressure type	Pressure change over time
*P1*	Proportional	Constant
*P2*	Proportional	Decreasing
*P3*	Proportional	Increasing
*F1*	Fixed	Constant
*F2*	Fixed	Decreasing
*F3*	Fixed	Increasing

For each scenario pressure was imposed at low, medium, and high levels on populations starting both far (*N*_1_ = 500) and at carrying capacity (*N*_1_ = 1000). Full details of scenarios in Table S2.

### Detection of decline-curve dynamics

Before searching for differences in decline curves within a simulated population, we smoothed the time-series using generalized additive models (GAMs; Wood 2006), in order to avoid picking up fluctuations resulting from stochastic, environmental variation. Smoothed abundance data are a more reliable indicator of population change compared to unsmoothed data (Porszt et al. 2012). GAMs are also an improvement over other trend analysis techniques (such as linear regression), as they allow the change in mean abundance to be represented by any smoothed curve shape that best-fits the data (Fewster et al. 2000; though see Soldaat et al. 2007). The degree of smoothness of the GAM was estimated automatically as part of the model fitting using the generalized cross validation (GCV) method, constrained at one less than the total length of the time-series (Fewster et al. 2000; Fedy and Doherty 2011). To reduce overfitting of the data, we included a penalty for each additional degree of freedom within the model by increasing the gamma parameter of the model to Wood's (2006) suggested value of 1.4. The GAM error distribution was left as default Gaussian.

As in recent population time-series analyses, (Siriwardena et al. 1998; Fewster et al. 2000; Collen et al. 2009), we detected shifts in population dynamics based on switches in a smoothed trend's second derivative sign. As the simulated trends are nonparametric curves, the second derivatives were not available directly as mathematical expressions, so we calculated approximate second derivative values for the time-series algebraically, based on the rate of change of the smoothed population abundance at each time step (see example in Table 3.). We used switches in the rate of change (or second derivative sign; herein termed second derivative switch points – SPs) to discriminate between curve sections according to their transition in decline speed. If a SP was recorded as occurring 1 year before the population became extinct, the trend was only analyzed up to the year preceding extinction.

**Table 3:** Example of SP calculation based on the rate of change between population counts

Year	1	2	3	4	5	6
Population count	50	60	75	70	60	45
Change	NA	10	15	−5	−10	−15
Rate of change	NA	NA	5	−20	−5	−5
SP presence	NA	NA	0	1	0	0

Change in population count was calculated by taking the yearly difference between counts, rate of change was calculated by taking the difference in change in counts and SPs were identified when the rate of changed switched from positive to negative.

To confirm that the SPs were associated with real changes in abundance driven by external pressure and not due to environmental stochasticity, we recalculated SPs across 100 simulated time-series with similar demographic properties under the same pressure scenarios as the focal time-series. We simulated the time-series by generating new population counts for each year based on the random normal distribution (with mean equal to the smoothed count for that year and standard deviation equal to the 95% confidence interval of the smoothed model fit) and defined the SPs as the years which were detected most frequently, out of all the time-series.

We applied the second derivative test (Larson et al. 1990) to determine the concavity of the curve between SPs. We identified a curve as “concave” (curved inwards) if its second derivative values were positive, and “convex” (curved outwards) if its second derivative values were negative. Before fitting functions to the data, we tested whether a population time-series section was significantly declining using a linear regression (with α = 0.05). We then determined the particular decline-curve type for each SP-delimited section longer than five data points (which we picked as an arbitrary cut-off) by fitting linear, quadratic, and exponential models to the data (detailed in Table 4), roughly corresponding to the curve types proposed by Mace et al. (2008). Specifically, we fitted the exponential model using a “Self-starting asymptotic exponential” function in R (“SSasymp”), whereas the others were fit manually using the formulae in Table 4. If a section was humped, concave or humped, convex, but was not significantly declining, we assessed the significance of its declining tail alone. If this was significant, we classed the whole section as declining. We determined the best-fitting function using a multimodel inference approach (Burnham and Anderson 2004), based on the model's Aikaike's Information Criterion (AIC; Akaike 1973), which we corrected for small sample size (AIC; eq. S1; Sugiura 1978) to avoid overfitting (i.e., when *n*/*k* < 40; *n* = sample size and *k* = number of parameters; Burnham and Anderson 2004). We chose the model with lowest AICc (based on a threshold of Δ AICc > 4; Burnham and Anderson 2004) as the one which best represented the declining trend. We relaxed the best-fit threshold to less than 4 when the model with the lower AICc was the simpler model (i.e., we chose the model with the least parameters if the difference between models was less than 4). If the number of data points within a declining section was two less than the number of parameters within the fitted model, then it was not possible to compute AICc, and we used ΔAIC to compare model fits. We tested the robustness of the results for each scenario by applying the steps described within this section to 1000 time-series generated under the same conditions.

**Table 4:** Model equations describing different decline-curve shapes

Model	Curve-shape distinction	Formula	Parameters
Linear	No concavity	*N*_*i*_ = *m*_1_*T*_*i*_ + *c*	*N*_we_ represents population size at time *i*, *T*_we_ represents year *i*, *c* represents the model intercept at *T*_we_ = 0, *m*_1_ represents the model slope.
Quadratic	Can be concave or convex. Quadratic concave curves can be distinguished from exponential concave curves as their rate of decline continues to decrease in a constant manner, leading to a right-hand side vertical asymptote.	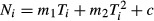	*N*_we_ represents population size at time *i*, *T*_we_ represents year *i*, *c* represents the model intercept at *T*_we_ = 0, *m*_1_ and *m*_2_ represent different model slopes.
Exponential	In exponential concave declines, decline rate slows as the population reduces in size, leading to a right-hand side horizontal asymptote.	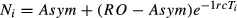	*N*_we_ represents population size at time *i*, *Asym* represents the horizontal asymptote of the model, *RO* represents the intercept at *T*_we_ = 0, *lrc* represents the model constant (i.e., decay rate).

### Time-series degradation analysis

The simulations involve perfect datasets that are unrepresentative of real world population data. We therefore degraded the simulated datasets to investigate how well more realistic data could be expected to retain a signal of pressure type identified in decline dynamics. Populations of medium-speed life-history species, starting at a population size close to *K* were simulated under increasing fixed (*F*3) and constant proportional (*P*1) pressure across a set of data quality degradation scenarios as follows:

- A shortening of time-series length in increments of 5 years, starting from 25 years: (a) either side of the onset of pressure; (b) only following the onset of pressure; and (c) only preceding the onset of pressure. For the last scenario, the years following the onset of pressure were reduced to two. This was not possible for (a) and (b) due to the minimum data requirement for second derivative calculation.
- A decrease in the frequency of population counts using gaps of: 1, 2, 3, 5, and 8 years between counts, linearly interpolating monitoring gaps using the R function “interpNA” (“timeSeries” package).
- Added observation error by resampling yearly population counts from a truncated normal distribution with mean equal to the population count for that year, and standard deviation (SD) equal to the SD of counts for that year across all 1000 simulated time-series, multiplied by 1.5, 2, or 2.5 (depending on level of error).

All degradation scenarios were repeated 1000 times. In order to determine the likelihood of identifying false positives, we tested our method on population simulations with the same demographic characteristics as the above time-series (i.e., with medium life-history speed parameters, starting close to *K*) but without any external pressure or degradation (herein termed null models).

### Application to wildlife populations

**Figure 3:**
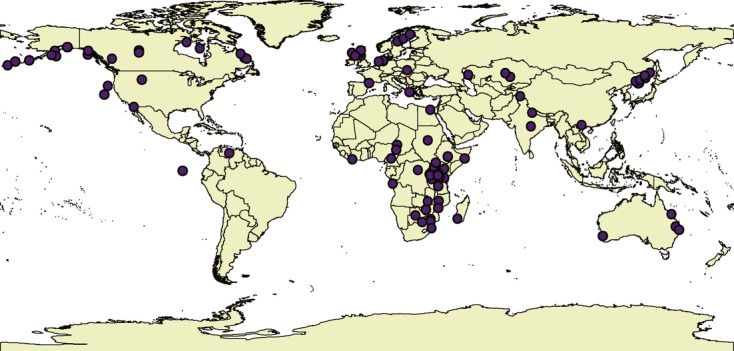
Location of mammal populations upon which we apply our decline-curve identification methods (*n* = 124).

In order to assess whether our methods could be applied to wildlife population time-series, we selected 124 mammal populations (Fig. 3) from the 1010 mammal population time-series in the LPI database. These represent 57 species spanning nine orders, and have the following attributes:

1. A minimum of three raw data points, which spanned a total of more than 5 years, and a gap of <8 years between data points.
2. Data were only collected from approximately 1900 onwards (data spanned between 1900 and 2010).
3. One or more threats attributed to the decline were recorded in the database, which were subsequently confirmed and updated by examination of the original data source.
4. Time-series were based on either full population counts or based on model population estimates.
5. Time-series were significantly (*P* < 0.05) decreasing or non-significantly increasing (*P* > 0.05) over time (based on linear regression).
6. Time-series had low environmental stochasticity and observation error (assessed by using time-series with small 95% CI; populations with highly stochastic fluctuations were excluded if the total reduction in population size was less than the difference between the upper and lower 95% CIs).

We refine the method for detecting decline-curve dynamics (see above) by adding the following steps when examining wildlife populations:

- Non-normality of the raw time-series data was accounted for by fitting a univariate generalized linear model (GLM) with poisson errors to the time-series with respect to time. If data were overdispersed, we used a quasipoisson link within subsequent GAM fitting.
- We set the upper degree of smoothness in each GAM to 0.3 times the length of the time-series (which represents a good trade-off between complexity and smoothness; Fewster et al. 2000; Fedy and Doherty 2011) and decreased smoothness to the lowest possible level, by decrements of one.
- Frequency of SP-identification across years was calculated based on the SPs identified from each smoothing level simulation. SPs years are those in which the frequency of SP-detection was greater than the upper 99.99% confidence interval around the median SP frequency. If none of the SP frequencies occurred at a frequency higher than this, then the choice of the most important SPs was from visual inspection of the dynamics. Specifically, where there were a few, proximate SP years, the year with highest SP support was designated as the SP for that period. In cases where proximate years had the same frequency of SP support, only the first year of the series was designated a SP.
- We tested whether the SP-delimited section was significantly declining (*P* < 0.05) by fitting a linear regression to the time-series section. If only part of the section was declining (e.g., in quadratic convex declines), we only fit the linear regression over this particular section.
- A jackknife analysis was used to derive a confidence limit around the best-fit decline-curve function.

Finally, we compared the pressure type which we hypothesize to be affecting the population (based on its decline curve) with information on the reported threatening process affecting each population. If a population was affected by more than one threat, then we recorded the decline curves under each threat type. We excluded climate change from the analysis as it was reported for only one population.

## Results

### Detection of decline-curve dynamics

**Figure 4:**
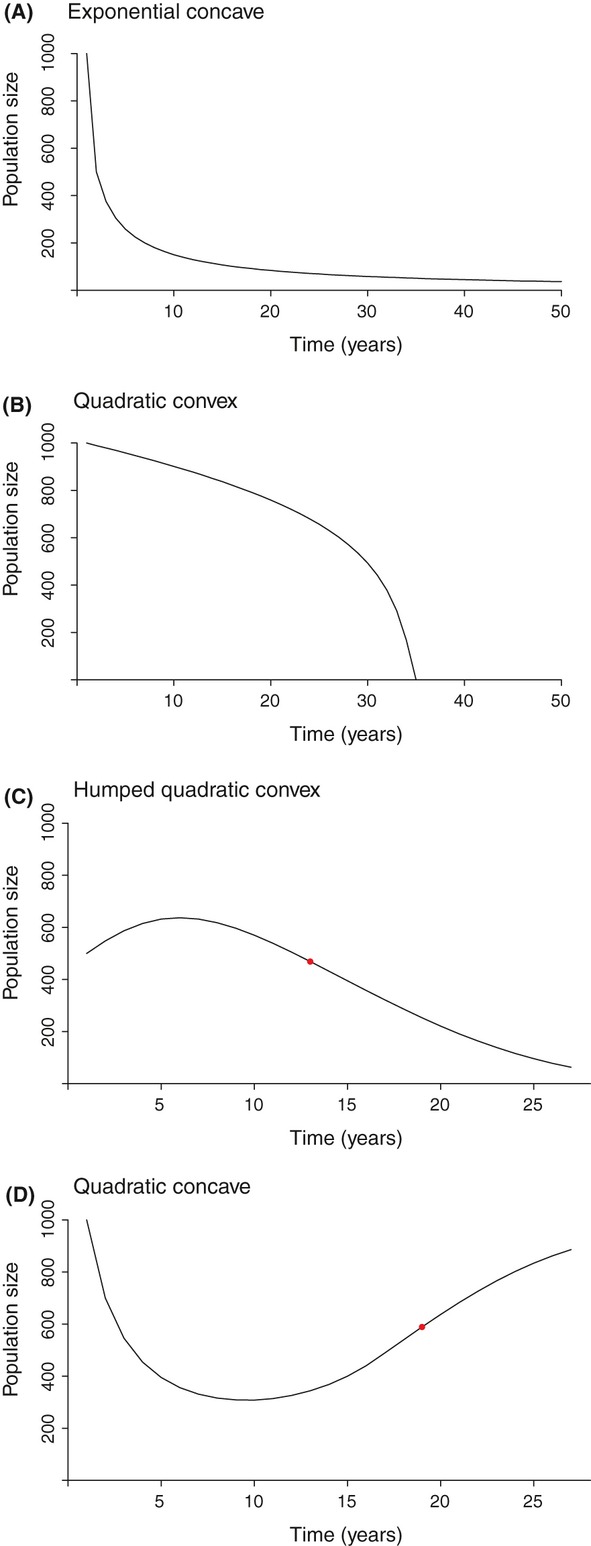
Decline patterns found in wildlife populations, associated with (A) simulated constant proportional pressure, (B) increasing fixed pressure (C) increasing proportional pressure starting far from *K,* and (D) decreasing proportional pressure. Red dots represent switch point (SP) locations. Where applicable, each graph title corresponds to the best-fit function of the first SP-delimited section.

Simulations of mammal populations under a range of pressure regimes indicated that only two pressure types led to a consistent response across all life-history speeds, starting population sizes, and pressure intensities tested in this study (Fig. 4A–B; details in Table S2 and S3). Specifically, scenarios of constant, proportional pressure (*P*1) and increasing fixed pressure types (*F*3) correspond to: exponential, concave; and quadratic, convex decline curves, respectively.

In addition to these robust associations we detected the following easily identifiable patterns (Fig. 4C–D; details in Table S2 and S3):

- Increasing proportional pressure types (*P*3) on population simulations which start far from *K* consistently result in quadratic, humped convex decline curves followed by concave declines.
- Decreasing proportional pressure types (*P*2) are consistently associated with concave declines; however, the algebraic function which describes the declining curve section is not consistent across scenarios. If pressure decreases at a fast enough rate, then this can be identified by a final, upwards turn in the curve.

### Time-series degradation analysis

All time-series degradations from constant proportional pressure (*P*1) were best-fit by exponential, concave curves at a significantly higher frequency than in the null models (in which pressure was not imposed). The only exception to this occurred when the time-series was shortened to 2 years prior to the onset of pressure, and the quadratic, concave function was best-fit (Table 5 and S4; Two-sample test for equality of proportions, χ^2^ = 0.76, df = 1, *P* = 0.38). Constant proportional pressure (*P*1) was more likely to result in exponential decline curves than in quadratic or linear declines across all time-series length degradation scenarios, whereas in scenarios where monitoring frequency was decreased and observation error increased, this was not always the case (Table S5.). For instance, when there were gaps of more than 2 years between monitoring (but less than five), there was no significant difference between the frequencies at which an exponential concave curve best-fit the decline compared to a quadratic, concave curve. Where the gap in monitoring was more than 5 years, an exponential curve was identified at a significantly lower frequency than a quadratic curve. In all scenarios with observation error, the decline curves were diagnosed as quadratic concaves at a significantly higher rate compared with exponential concave curves. The only scenario in which low, constant, proportional pressure was significantly more likely to be diagnosed as having a convex shape over a concave, was when there was a 8 year gap in monitoring (Table S6; Two-sample test for equality of proportions, *χ*^2^ = 0.02, df = 1, *P* = 0.90).

**Table 5:** Best-fit function and concavity of best-fit decline curves caused by low, constant proportional pressure (*P*1), under different scenarios of time-series quality degradation

		Best-fit function (out of 1000 simulations)			Concavity (out of 1000 simulations)	
			
Degradation type	Specific degradation	Linear (%)	Quadratic (%)	Exponential (%)	Concave (%)	Convex (%)
Years either side of pressure	25 (None)	6.9	42.8	50.3	88.2	11.8
Years either side of pressure	20	6.6	37.4	56	89.7	10.3
Years either side of pressure	15	5.6	35.9	58.5	89.4	10.6
Years either side of pressure	10	8.8	23.3	67.9	90.8	9.2
Years either side of pressure	5	0.1	43	56.9	100	0
Years after pressure	20	7.4	38	54.6	88.2	11.8
Years after pressure	15	4.4	36.7	58.9	87.4	12.6
Years after pressure	10	8.9	28.4	62.7	87.6	12.4
Years after pressure	5	5.5	39.1	55.4	83.8	16.2
Years before pressure	20	9.9	38.4	51.7	90.5	9.5
Years before pressure	15	4.6	42	53.4	90.7	9.3
Years before pressure	10	5.8	38.6	55.6	93.3	6.7
Years before pressure	5	6.8	36.4	56.8	97.9	2.1
Years before pressure	2	18.8	68.9	9.6	59.5	40.5
Years between monitoring	1	6.2	39.2	54.6	88.8	11.2
Years between monitoring	2	7.8	43.4	48.8	88.7	11.3
Years between monitoring	3	9.5	43	47.5	88.3	11.7
Years between monitoring	5	21.3	44	34.7	67.6	32.4
Years between monitoring	8	21.5	61.9	16.6	50.2	49.8
Magnitude of observation error	1	15.8	51.5	32.3	78.7	21.3
Magnitude of observation error	1.5	12.5	62.5	24.4	78.3	21.7
Magnitude of observation error	2	10.8	65.4	21.9	76.7	23.3
Magnitude of observation error	2.5	12.8	72	13.3	70.8	29.2

Quadratic convex declines were consistently identified as best-fit curve in response to increasing, fixed pressure (*F*3), regardless of any degradation in time-series quality (Table 6). Indeed, quadratic convex decline curves were identified across all such scenarios of degraded time-series affected by increasing fixed pressure at a significantly higher frequency than in the null population models (Table 7 and Table S7), and compared with linear and exponential functions (Tables S8 and S9).

**Table 6:** Best-fit function and concavity of best-fit decline curves in medium life-history speed populations affected by increasing, fixed pressure (*F*3), under different scenarios of time-series quality degradation

		Best-fit function (out of 1000 simulations)			Concavity (out of 1000 simulations)	
			
Degradation type	Specific degradation	Linear (%)	Quadratic (%)	Exponential (%)	Concave (%)	Convex (%)
Years either side of pressure	25 (None)	1.7	98.3	0	1	99
Years either side of pressure	20	15.5	84.5	0	5.8	94.2
Years either side of pressure	15	14.4	85.5	0.1	6.6	93.4
Years either side of pressure	10	13.7	86	0.3	5.5	94.5
Years either side of pressure	5	4.2	95.5	3	7	93
Years after pressure	20	15.4	84.5	0.1	7.8	92.2
Years after pressure	15	16.9	82.8	0.3	8.8	91.2
Years after pressure	10	16.9	82.7	0.4	6.2	93.8
Years after pressure	5	4.9	94	1.1	10.3	89.7
Years before pressure	20	12.5	87.5	0	3.7	96.3
Years before pressure	15	11.1	88.8	0.1	5.4	94.6
Years before pressure	10	12.5	87.5	0	5.5	94.5
Years before pressure	5	11.6	88.4	0	3.3	96.7
Years before pressure	2	12.4	87.5	0.1	5.6	94.4
Years between monitoring	1	13.9	85.9	0.2	8.1	91.9
Years between monitoring	2	14.3	85.5	0.2	6.5	93.5
Years between monitoring	3	10.1	89.8	0.1	6.1	93.9
Years between monitoring	5	9.9	89.8	0.3	5.4	94.6
Years between monitoring	8	5.8	94.2	0	0.7	99.3
Magnitude of observation error	1	4.3	95.6	0.1	8	92
Magnitude of observation error	1.5	4.7	95	0.3	7.6	92.4
Magnitude of observation error	2	3.7	95.8	0.5	11.2	88.8
Magnitude of observation error	2.5	3.8	96.1	0.1	11	89

**Table 7:** Best-fit function and concavity of null model population simulations with medium life-history speed characteristics, starting close to *K*

Curve type	Best-fit out of 1000 simulations (%)
Linear	24.5
Quadratic	67.1
Exponential	8.4
Concave	52.1
Convex	47.9

### Application to wildlife populations

**Figure 5:**
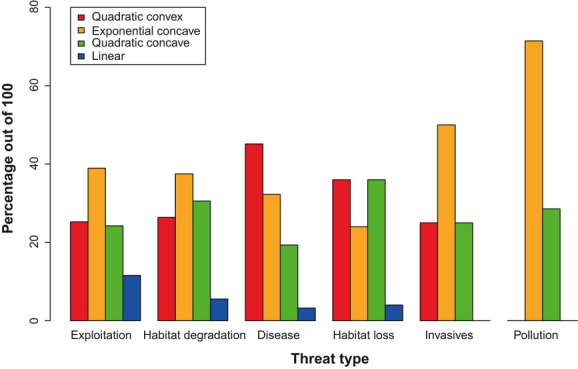
Distribution of decline curves according to threatening process (based on percentage identified out of all decline curves ascribed with the same threat type; N.B. multiple threats were reported for each population time-series). *N* = 95 decline curves affected by exploitation, 72 by habitat degradation or alteration, 31 by disease, 25 by habitat loss, eight by invasive species or genes, and seven by pollution.

We identified 159 decline curves in 124 population time-series. Significantly more were concave (62.3%) than convex (28.3%; Two-sample test for equality of proportions, χ^2^ = 35.65, df *=* 1, *P* < 0.001). 60.6% of all concave decline curves were best described by exponential functions and the remainder best-fit by quadratic functions. Although quadratic convex declines were the next most frequently diagnosed decline type, these were not identified significantly more frequently than quadratic concave declines (24.5%; Two-sample test for equality of proportions, χ^2^ = 0.40, df = 1, *P* = 0.52). Only 9.4% of declines were linear. When we categorized decline curves according to reported threat type (with multiple threats per decline, *n* = 238; Fig. 5), exponential concave declines were most prevalent in populations affected by exploitation, habitat degradation, invasive species, and pollution. Within disease-affected populations, we identified four more quadratic convex declines than exponential concave declines, and in populations affected by habitat loss, we identified three more convex declines. Quadratic convex and concave declines occurred in approximately the same proportions across all threat types, with the exception of disease-affected populations where quadratic convex declines were much more frequent, and amongst those affected by pollution, where convex declines were not present at all. Linear declines were least common across all threat categories.

## Discussion

Reductions in population size are often recorded, but on their own they can be weak indicators of the urgency or importance of conservation interventions. This study illustrates how determining the dynamics of a population decline can inform decisions about whether efforts for a population's conservation are urgent and should be prioritized.

### Validation of decline-curve associations

These analyses explore the idea that different threatening processes may lead to different population decline dynamics that should be diagnosable from good quality monitoring data. Based on simulations, we found that only two of the decline curves proposed by Mace et al. (2008) were consistently attributable to the same pressure types across different population life-history speeds, proximity to carrying capacity, and pressure intensities. These consistent decline-curve types are caused by constant, proportional pressure (*P*1) and increasing, fixed pressure (*F*3), and, respectively, result in exponential, concave and quadratic, convex population decline curves. We show that these curves are the result of different pressure regimes and do not just arise by chance. Under more realistic scenarios of wildlife population data collection, quadratic convex curves caused by increasing, fixed pressure (*F*3) appear to be extremely robust to simulated deteriorations in data quality. In contrast, the dynamical responses of populations to constant proportional pressure (*P*1) are vulnerable to degrading data quality, and have a tendency to switch from exhibiting exponential, concave declines to quadratic, concave declines, especially when there is little monitoring in advance of the pressure, when monitoring intensity is sparse, or there are large observation errors. This change in best-fit function may be a consequence of the quadratic function being mathematically simpler than the exponential, so it is more likely to be identified in scenarios where the time-series is less well documented. Despite the general decline-curve associations which we detect in our analysis, when pressure is very weak such dynamical patterns may become obscured by population fluctuations due to environmental and demographic stochasticity (Morris and Doak 2002), the influence of intrinsic factors such as density dependence (Lomolino and Channell 1995; Rodriguez 2002; Akçakaya et al. 2006) or observation error (Hilborn and Mangel 1997).

In addition to the above, robust decline-curve associations, we find a few easily identifiable trends which can be used to inform understanding of how the pressure acting upon a population is changing over time. These include the detection of (a) a final concave, upwards turn, associated with decreasing pressure; and (b) a hump in the time-series, followed by a concave decline, which is associated with increasing proportional pressure acting upon a population that is far from carrying capacity. The identification of a final, upwards turn can be explained by the pressure reducing so much that it ceases to limit population growth, with resulting recovery. The presence of a hump in response to increasing, proportional pressure suggests the pressure is initially weak enough to allow population growth, but once it crosses a higher intensity threshold the population starts to decline. The decline curve switches from convex to concave as population size diminishes and the pressure has a proportionally smaller effect over time (even though it is still increasing). We do not find any clear population dynamical responses to constant, fixed or decreasing, fixed pressure, which may be due to the nonlinear impact which a fixed removal of individuals has on populations. Indeed, the impact of fixed pressure upon a wildlife population will vary depending on how fast as species can recover (based on its intrinsic rate of growth) and its proximity to carrying capacity (where a removal of individuals may be actually be beneficial when a population is near *K*).

### Decline curves in wildlife populations

A search for consistent decline-curve patterns and specific threat responses in noisy wildlife population time-series identified three principal decline-curve types: quadratic convex, quadratic concave, and exponential concave. Overall, exponential concave declines are the most common in our dataset, suggesting that in most cases, populations are affected by proportional pressure that is decreasing in intensity as populations decrease in abundance. As quadratic convex declines result in the most rapid population reductions, it is understandable that these should be relatively rare within our dataset of well-monitored mammals. Quadratic concave declines are equally uncommon, perhaps because the pressures featured in this dataset have not yet decreased to a level which allows population recovery. At the most basic level, convex decline curves can be associated with increasing rates of decline in response to pressure and concave curves with decreasing decline rates. The distinction between exponential and quadratic concave curves is harder to interpret given the imperfect nature of wildlife data collection, nevertheless, if the algebraic function can be distinguished this would provide further insight into whether pressure is decreasing because it has a proportional effect (and is therefore decreasing with decreasing population size) or if it is directly decreasing over time.

### Relevance to the IUCN Red List

Identifying differences in the concavity of population declines could provide an important refinement to the classification of threatened species. Although “high population decline-rate” is already a key criterion in the International Union for Conservation of Nature's (IUCN) system for classifying threatened species (Mace and Lande 1991; IUCN Standards and Petitions Subcommittee 2013), it is only based on “percentage loss” and does not include any information on whether the rate of loss is changing over time. The addition of decline concavity to the process of ascribing extinction-risk status may enable us to identify species experiencing accelerating population declines, potentially classifying them in a higher threat category. Regardless, the reliability of the decline concavity assessment could be used to allocate a higher priority for conservation attention. This could also be used as a method for prioritizing which populations of a threatened species require more urgent conservation action, however, such decisions should also be considered in light of the feasibility and cost of their recovery (e.g., as described in Joseph et al. 2009). The focus on decline-curve shape could also be extended to the context of aggregate biodiversity trends (e.g., Collen et al. 2009), where it could provide an additional method for classifying decline severity. Given that funding for conservation is limited (as discussed in MacKenzie 2009), statistically analyzing population time-series for signals of convex declines represents a potential cost-effective method for conservation decision making.

### Lack of association with threats

Contrary to Mace et al.'s (2008) proposal, which links four distinct decline trajectories with four broad categories of threatening processes, we do not find any clear and consistent associations with particular threat types. Our inability to match different decline curves to specific threats, suggests that rather than using them as a way to determine the particular cause of decline, they could better be used to infer the type of pressure which is influencing its dynamics. Given that it is currently so common for drivers of biodiversity loss to act at the same time upon wildlife populations (Brook et al. 2008; Acevedo-Whitehouse and Duffus 2009; Laurance and Useche 2009), we propose using this method to determine the nature of the pressure affecting a population (i.e., if it is increasing, decreasing, or having a constant, proportional effect). Such information could be used to identify the principal threatening process out of a range of reported threats with different pressure intensities and dynamics. Obtaining a better understanding of the major threat affecting a population will be critical for more effective, directed conservation action.

### Methodological issues

The choice of the logistic model to explore the response of wildlife populations to different pressure regimes may be perceived as a potential limitation of this study. Albeit widely used, it has a number of recognized failings that we do not address, including the assumptions that: (1) growth is linearly related to population density; (2) carrying capacity is constant; (3) the rate of population change responds immediately to variations in density; and (4) there is no population structure (Turchin 2003; Clark et al. 2010). While alternatives to the logistic model exist, which account for these shortcomings (e.g., the theta-logistic or matrix models; Leslie 1945; Gilpin and Ayala 1973) we chose to use the simplest unstructured discrete time model for single species dynamics in order to develop a method which could be applied to any population time-series, irrespective of data quality. It is possible that by specifying the section of the population upon which pressure is acting we might identify a population response which is stronger or weaker than those detailed above, depending on the contribution of that particular section to overall population growth (Caswell 2001), however, the population's general decline pattern should remain the same. It is also likely that a population's decline-curve dynamics will remain consistent across the effects of nonlinear density dependence (e.g., in large-mammal species, where density-dependence is mainly experienced close to carrying capacity and almost nonexistent at lower densities; Fowler 1981), as it will only be possible to detect declines if the pressure imposed is stronger than a population's natural tendency to increase, whether it is affected by density-dependence or not. Decline-curve dynamics should also remain constant if the population is affected by competition or predation by neighboring species or by multiple threats, as long as the pressure imposed by the principal threatening process is strong enough to leave a distinct signal.

A further methodological caveat lies in the identification of declines based on statistical hypothesis testing, and best-fit decline curves according to a multimodel inference framework. Both steps require sufficient evidence (e.g., a sufficient number of data points) to calculate, which may not always be available (as critiqued in Nichols and Williams 2006). Furthermore, we base decline significance tests using a Type I error rate (α) of 0.05, which is a widely accepted arbitrary cut-off that may prevent the detection of declines that do not quite fall within this criterion (causing a Type II error; discussed in Di Stefano 2003), and could result in potential performance failings (e.g., Di Stefano 2001). We also only assessed decline significance in relation to the first data point of the time-series section in focus, which does not provide any information on its decline relative to historical baselines of population abundance (found to be the most useful aspect defining the reliability of decline indicators; Porszt et al. 2012). Finally, we used a minimum ΔAICc of 4 in order to choose the best-fit model for each decline-curve type, which may be too high to distinguish more subtle differences in declines. Further studies could examine the level of statistical confidence which is required (e.g., through power analyses) in order to categorize population dynamics into different declining curve sections. The initial test for decline significance may benefit from a more precautionary approach, which would increase the risk of detecting false positives. Following this step, a more detailed examination of decline-curve type would identify the more rapid population declines.

## Supporting Information

**Table S1.** Full description of pressure scenario imposed on populations with fast, medium, and slow life-history speeds. *N*_1_ represents starting population size.

**Table S2.** Deterministic curve shapes produced by different scenarios of proportional pressure on fast, medium, and slow life-history speed population models. The section of a decline curve before the switch point (SP) is indicated by “A” and the following section by “B”.

**Table S3.** Deterministic curve shapes produced by different scenarios of fixed pressure on fast, medium, and slow life-history speed population models. The section of a decline curve before the switch point (SP) is indicated by “A” and the following section by “B”.

**Table S4.** Chi-square results from two-sample test for equality of proportions between the best-fit results in null population models and those in low, constant, proportional pressure scenarios (*P*1). Significant differences at α = 0.05 are indicated by a star.

**Table S5.** Chi-square results from two-sample test for equality of proportions between best-fit frequencies for each degradation scenario in populations with low, constant, proportional pressure (*P*1). Significant differences at α = 0.05 are indicated by a star.

**Table S6.** Chi-square results from two-sample test for equality of proportions between the concavity frequencies diagnosed for each degradation scenario in populations with low, constant, proportional pressure (*P*1). Significant differences at α = 0.05 are indicated by a star.

**Table S7**. Chi-square results from two-sample test for equality of proportions between the best-fit results in null population models and those in increasing fixed pressure scenarios (*F*3). Significant differences at α = 0.05 are indicated by a star.

**Table S8.** Chi-square results from two-sample test for equality of proportions between best-fit frequencies for each degradation scenario in populations with increasing, fixed pressure (*F*3). Significant differences at α = 0.05 are indicated by a star.

**Table S9.** Chi-square results from two-sample test for equality of proportions between the concavity frequencies diagnosed for each degradation scenario in populations with increasing, fixed pressure (*F*3). Significant differences at α = 0.05 are indicated by a star.

## References

[b1] Acevedo-Whitehouse K, Duffus ALJ (2009). Effects of environmental change on wildlife health. Philos. Trans. R. Soc. Lond. B Biol. Sci.

[b2] Akaike H (1973). Information theory and an extension of the maximum likelihood principle.

[b3] Akçakaya HR, Butchart SHM, Mace GM, Stuart SN, Hilton-Taylor C (2006). Use and misuse of the IUCN Red List Criteria in projecting climate change impacts on biodiversity. Glob. Change Biol.

[b4] Allee WC (1931). Animal Aggregations. A Study in General Sociology.

[b5] Balmford A, Green RE, Jenkins M (2003). Measuring the changing state of nature. Trends Ecol. Evol.

[b6] Boakes EH, Mace GM, McGowan PJK, Fuller RA (2009). Extreme contagion in global clearance. Proc. Biol. Sci.

[b7] Brook BW, Sodhi NS, Bradshaw CJA (2008). Synergies among extinction drivers under global change. Trends Ecol. Evol.

[b8] Burnham KP, Anderson DR (2004). Multimodel inference: understanding AIC and BIC in model selection. Sociol. Methods Res.

[b9] Butchart SHM, Walpole M, Collen B, Scharlemann A, van Strien JPW, Almond REA (2010). Global biodiversity: indicators of recent declines. Science.

[b10] Caswell H (2001). Matrix Population Models: construction, analysis and interpretation.

[b11] Ceballos G, Ehrlich PR (2002). Mammal population losses and the extinction crisis. Science.

[b12] Clark F, Brook BW, Delean S, Akçakaya HR, Bradshaw CJA (2010). The theta-logistic is unreliable for modelling most census data. Methods Ecol. Evol.

[b13] Collen B, Loh J, Holbrook S, McRae L, Amin R, Baillie JEM (2009). Monitoring change in vertebrate abundance: the Living Planet Index. Conserv. Biol.

[b14] Convention on Biological Diversity (2002).

[b15] Courchamp F, Clutton-Brock TH, Grenfell B (1999). Inverse density dependence and the Allee effect. Trends Ecol. Evol.

[b16] Courchamp F, Angulo E, Rivalan P, Hall RJ, Signoret L, Bull L (2006). Rarity value and species extinction: the anthropogenic Allee effect. PLoS Biol.

[b17] Di Stefano J (2001). Power analysis and sustainable forest management. For. Ecol. Manage.

[b18] Di Stefano J (2003). How much power is enough? Against the development of an arbitrary convention for statistical power calculations. Funct. Ecol.

[b19] Fedy BC, Doherty KE (2011). Population cycles are highly correlated over long time series and large spatial scales in two unrelated species: greater sage-grouse and cottontail rabbits. Oecologia.

[b20] Fewster RM, Buckland ST, Siriwardena GM, Baillie SR, Wilson JD (2000). Analysis of population trends for farmland birds using generalized additive models. Ecology.

[b21] Fowler CW (1981). Density dependence as related to life history strategy. Ecology.

[b22] Fowler CW (1988). Population dynamics as related to rate of increase per generation. Evol. Ecol.

[b23] Gaillard J-M, Festa-Bianchet M, Yoccoz NG, Loson A, Togo C (2000). Temporal variation in fitness components and population dynamics of large herbivores. Annu. Rev. Ecol. Syst.

[b24] Getz WM, Haight RG (1989). Population harvesting: demographic models of fish, forest and animal resources.

[b25] Gilpin ME, Ayala FJ (1973). Global models of growth and competition. Proc. Natl Acad. Sci. USA.

[b26] Hilborn R, Mangel M (1997). The ecological detective: confronting models with data.

[b27] Hjerne O, Hansson S (2001). Constant catch or constant harvest rate? The Baltic Sea cod (*Gadus morhua* L.) fishery as a modelling example. Fish. Res.

[b28] IUCN Standards and Petitions Subcommittee (2013).

[b29] Jones KE, Bielby J, Cardilllo M, Fritz SA, O'Dell J, Orme CDL (2009). PanTHERIA: a species-level database of life history, ecology, and geography of extant and recently extinct mammals. Ecology.

[b30] Joseph LN, Maloney RF, Possingham HP (2009). Optimal allocation of resources among threatened species: a project prioritization protocol. Conserv. Biol.

[b31] Larson R, Hostetler RP, Edwards BH (1990). Calculus.

[b32] Laurance WF, Useche D (2009). Environmental synergisms and extinctions of tropical species. Conserv. Biol.

[b33] Leslie PH (1945). On the use of matrices in population mathematics. Biometrika.

[b34] Loh J, Green RE, Ricketts T, Lamoreux J, Jenkins M, Kapos V (2005). The Living Planet Index: using species population time series to track trends in biodiversity. Philos. Trans. R. Soc. Lond. B Biol. Sci.

[b35] Lomolino MV, Channell R (1995). Splendid isolation: patterns of geographic range collapse in endangered mammals. J. Mammal.

[b36] Mace GM, Lande R (1991). Assessing extinction threats: towards a reevaluation of IUCN threatened species categories. Conserv. Biol.

[b37] Mace GM, Collar NJ, Gaston KJ, Hilton-Taylor C, Akçakaya HR, Leader-Williams N (2008). Quantification of extinction risk: IUCN's system for classifying threatened species. Conserv. Biol.

[b38] MacKenzie DI (2009). Getting the biggest bang for our conservation buck. Trends Ecol. Evol.

[b39] McRae L, Collen B, Deinet S, HIll P, Loh J, Baillie JEM, Price V (2012). The Living Planet Index.

[b40] Morris WF, Doak DF (2002). Count-based PVA: incorporating density dependence, demographic stochasticity, correlated environments, catastrophes and bonanzas. Page 100 Quantitative conservation biology.

[b41] Myers RA, Barrrowman NJ, Hutchings JA, Rosenberg AA (1995). Population dynamics of exploited fish stocks at low population levels. Science.

[b42] Nichols JD, Williams BK (2006). Monitoring for conservation. Trends Ecol. Evol.

[b43] Pople T, Grigg G (1999).

[b44] Porszt EJ, Peterman RM, Dulvy NK, Cooper AB, Irvine JR (2012). Reliability of indicators of decline in abundance. Conserv. Biol.

[b45] R Development Core Team (2012). R: a language and environment for statistical computing.

[b46] Rodriguez JP (2002). Range contraction in declining North American bird populations. Ecol. Appl.

[b47] Siriwardena GM, Baillie SR, Buckland ST, Fewster RM, Marchant JM, Wilson JD (1998). Trends in the abundance of farmland birds: a quantitative comparison of smoothed Common Birds Census indices. J. Appl. Ecol.

[b48] Soldaat L, Visser H, van Roomen M (2007). Smoothing and trend detection in waterbird monitoring data using structural time-series analysis and the Kalman filter. J. Ornithol.

[b49] Sugiura N (1978). Further analysis of the data by Akaike's Information Criterion and the finite corrections. Commun. Stat. Theory Methods.

[b50] Turchin P (2003). Complex population dynamics: a theoretical, empirical synthesis.

[b51] Verhulst PF (1838). Notice sur la loi que la population suit dans son accroisement. Correspondence Mathematique et Physique.

[b52] Wilson HB, Kendall BE, Possingham HP (2011). Variability in population abundance and the classification of extinction risk. Conserv. Biol.

[b53] Wood SN (2006). Generalized Additive Models: an introduction with R.

[b54] Yoccoz NG, Nichols JD, Boulinier T (2001). Monitoring of biological diversity in space and time. Trends Ecol. Evol.

