# Supplementary material for: A new method for identifying rapid decline dynamics in wild vertebrate populations
Source: Ecol Evol. 2013 Jun 14;3(7):2378–91. doi: 10.1002/ece3.596 (PMC3728972; doi:10.1002/ece3.596)
Supplement: Supplementary file 1 [file ece30003-2378-SD1.docx]

**Supplementary Information**

**Eq. S1.** $AICc=\left(2k-2\log(L)\right)+\left(\frac{\text{2k(k}+\text{1)}}{n-k-1}\right)$;

$AICc=\left( 2k-2log(L) \right)+ \frac{2k(k+1)}{n-k-1}$*k* = number of parameters, *L* =likelihood of the model given the data, and *n*= sample size.

**Table S1.** Full description of pressure scenario imposed on populations with fast, medium and slow life-history speeds. *N_1_* represents starting population size.

| **Scenario code category** | **Pressure type** | **Pressure change over time** | **Level** | **N_1_** | **Starting pressure (% or fixed loss)** | **Yearly change in pressure** | **Final pressure** |
| --- | --- | --- | --- | --- | --- | --- | --- |
| P1 | Proportional | Constant | Low | 500 and 1000 | 10 | 0 | 10 |
| P1 | Proportional | Constant | Medium | 500 and 1000 | 20 | 0 | 20 |
| P1 | Proportional | Constant | High | 500 and 1000 | 30 | 0 | 30 |
| P2 | Proportional | Decreasing | Slow | 500 and 1000 | 30 | -0.5 | 0 |
| P2 | Proportional | Decreasing | Medium | 500 and 1000 | 30 | -1 | 0 |
| P2 | Proportional | Decreasing | High | 500 and 1000 | 30 | -2 | 0 |
| P3 | Proportional | Increasing | Slow | 500 and 1000 | 5 | 0.5 | 70 |
| P3 | Proportional | Increasing | Medium | 500 and 1000 | 5 | 1 | 70 |
| P3 | Proportional | Increasing | High | 500 and 1000 | 5 | 2.5 | 70 |
| F1 | Fixed | Constant | Low | 1000 | 30 | 0 | 10 |
| F1 | Fixed | Constant | Medium | 1000 | 50 | 0 | 25 |
| F1 | Fixed | Constant | High | 1000 | 75 | 0 | 70 |
| F1 | Fixed | Constant | Low | 500 | 70 | 0 | 80 |
| F1 | Fixed | Constant | Medium | 500 | 80 | 0 | 100 |
| F1 | Fixed | Constant | High | 500 | 100 | 0 | 100 |
| F2 | Fixed | Decreasing | Slow | 1000 | 100 | -1 | 0 |
| F2 | Fixed | Decreasing | Medium | 1000 | 100 | -2 | 0 |
| F2 | Fixed | Decreasing | High | 1000 | 100 | -3 | 0 |
| F2 | Fixed | Decreasing | Slow | 500 | 80 | -1 | 0 |
| F2 | Fixed | Decreasing | Medium | 500 | 80 | -2 | 0 |
| F2 | Fixed | Decreasing | High | 500 | 80 | -3 | 0 |
| F3 | Fixed | Increasing | Slow | 1000 | 1 | 2 | 52 |
| F3 | Fixed | Increasing | Medium | 1000 | 1 | 5 | 130 |
| F3 | Fixed | Increasing | High | 1000 | 1 | 7 | 182 |
| F3 | Fixed | Increasing | Slow | 500 | 1 | 5 | 130 |
| F3 | Fixed | Increasing | Medium | 500 | 1 | 8 | 201 |
| F3 | Fixed | Increasing | High | 500 | 1 | 9 | 226 |

**Table S2.** Deterministic curve-shapes produced by different scenarios of proportional pressure on fast, medium and slow life-history speed population models. The section of a decline-curve before the switch point (SP) is indicated by “A” and the following section by “B”.

| **Pressure type** | **Pressure change over time** | ***r_max_*** | **Pressure level** | **N_1_** | **SP** | **Section** | **Shape** | **Best fit** |
| --- | --- | --- | --- | --- | --- | --- | --- | --- |
| Proportional | Constant | 0.1 | Low | Any | 0 | NA | Linear | Linear |
| Proportional | Constant | 0.2 | Low | Any | 0 | NA | Concave | Exponential |
| Proportional | Constant | 0.3 | Low | Any | 0 | NA | Concave | Exponential |
| Proportional | Constant | 0.1 | Medium | Any | 0 | NA | Concave | Exponential |
| Proportional | Constant | 0.2 | Medium | Any | 0 | NA | Concave | Exponential |
| Proportional | Constant | 0.3 | Medium | Any | 0 | NA | Concave | Exponential |
| Proportional | Constant | 0.1 | High | Any | 0 | NA | Concave | Exponential |
| Proportional | Constant | 0.2 | High | Any | 0 | NA | Concave | Exponential |
| Proportional | Constant | 0.3 | High | Any | 0 | NA | Concave | Exponential |
| Proportional | Increasing | 0.1 | Low | 1000 | 0 | NA | Concave | Exponential |
| Proportional | Increasing | 0.2 | Low | 1000 | 0 | NA | Concave | Exponential |
| Proportional | Increasing | 0.3 | Low | 1000 | 0 | NA | Concave | Exponential |
| Proportional | Increasing | 0.1 | Medium | 1000 | 1 | A | Convex | Quadratic |
| Proportional | Increasing | 0.1 | Medium | 1000 | 1 | B | Concave | Quadratic |
| Proportional | Increasing | 0.2 | Medium | 1000 | 0 | NA | Concave | Quadratic |
| Proportional | Increasing | 0.3 | Medium | 1000 | 0 | NA | Concave | Exponential |
| Proportional | Increasing | 0.1 | High | 1000 | 1 | A | Linear | Linear |
| Proportional | Increasing | 0.1 | High | 1000 | 1 | B | Concave | Quadratic |
| Proportional | Increasing | 0.2 | High | 1000 | 1 | A | Linear | Linear |
| Proportional | Increasing | 0.2 | High | 1000 | 1 | B | Concave | Quadratic |
| Proportional | Increasing | 0.3 | High | 1000 | 1 | A | Concave | Quadratic |
| Proportional | Increasing | 0.3 | High | 1000 | 1 | B | Convex | Quadratic |
| Proportional | Increasing | 0.1 | Low | 500 | 1 | A | Convex, hump | Quadratic |
| Proportional | Increasing | 0.1 | Low | 500 | 1 | B | Concave | Quadratic |
| Proportional | Increasing | 0.2 | Low | 500 | 1 | A | Convex, hump | Quadratic |
| Proportional | Increasing | 0.2 | Low | 500 | 1 | B | Linear | Linear |
| Proportional | Increasing | 0.3 | Low | 500 | 1 | A | Convex, hump | Quadratic |
| Proportional | Increasing | 0.3 | Low | 500 | 1 | B | Concave | Quadratic |
| Proportional | Increasing | 0.1 | Medium | 500 | 1 | A | Convex, hump | Quadratic |
| Proportional | Increasing | 0.1 | Medium | 500 | 1 | B | Concave | Quadratic |
| Proportional | Increasing | 0.2 | Medium | 500 | 1 | A | Convex, hump | Quadratic |
| Proportional | Increasing | 0.2 | Medium | 500 | 1 | B | Concave | Quadratic |
| Proportional | Increasing | 0.3 | Medium | 500 | 1 | A | Convex, hump | Quadratic |
| Proportional | Increasing | 0.3 | Medium | 500 | 1 | B | Concave | Quadratic |
| Proportional | Increasing | 0.1 | High | 500 | 1 | A | Convex, hump | Quadratic |
| Proportional | Increasing | 0.1 | High | 500 | 1 | B | Concave | Exponential |
| Proportional | Increasing | 0.2 | High | 500 | 1 | A | Convex, hump | Quadratic |
| Proportional | Increasing | 0.2 | High | 500 | 1 | B | Concave | Quadratic |
| Proportional | Increasing | 0.3 | High | 500 | 1 | A | Convex, hump | Quadratic |
| Proportional | Increasing | 0.3 | High | 500 | 1 | B | Concave | Quadratic |
| Proportional | Decreasing | 0.1 | Low | 1000 | 0 | NA | Concave | Exponential |
| Proportional | Decreasing | 0.2 | Low | 1000 | 0 | NA | Concave | Exponential |
| Proportional | Decreasing | 0.3 | Low | 1000 | 0 | NA | Concave | Exponential |
| Proportional | Decreasing | 0.1 | Medium | 1000 | 0 | NA | Concave | Quadratic |
| Proportional | Decreasing | 0.2 | Medium | 1000 | 0 | NA | Concave | Exponential |
| Proportional | Decreasing | 0.3 | Medium | 1000 | 0 | NA | Concave | Quadratic |
| Proportional | Decreasing | 0.1 | High | 1000 | 0 | NA | Concave | Quadratic |
| Proportional | Decreasing | 0.2 | High | 1000 | 0 | A | Concave | Quadratic |
| Proportional | Decreasing | 0.3 | High | 1000 | 0 | NA | Concave | Quadratic |
| Proportional | Decreasing | 0.1 | Low | 500 | 0 | NA | Concave | Exponential |
| Proportional | Decreasing | 0.2 | Low | 500 | 0 | NA | Concave | Exponential |
| Proportional | Decreasing | 0.3 | Low | 500 | 0 | NA | Concave | Exponential |
| Proportional | Decreasing | 0.1 | Medium | 500 | 0 | NA | Concave | Exponential |
| Proportional | Decreasing | 0.2 | Medium | 500 | 0 | NA | Concave | Exponential |
| Proportional | Decreasing | 0.3 | Medium | 500 | 0 | NA | Concave | Exponential |
| Proportional | Decreasing | 0.1 | High | 500 | 0 | NA | Concave | Quadratic |
| Proportional | Decreasing | 0.2 | High | 500 | 0 | NA | Concave | Quadratic |
| Proportional | Decreasing | 0.3 | High | 500 | 0 | NA | Concave | Exponential |

**Table S3.** Deterministic curve-shapes produced by different scenarios of fixed pressure on fast, medium and slow life-history speed population models. The section of a decline-curve before the switch point (SP) is indicated by “A” and the following section by “B”.

| **Pressure type** | **Pressure change over time** | ***r_max_*** | **Pressure level** | **N_1_** | **SP** | **Section** | **Shape** | **Best fit** |
| --- | --- | --- | --- | --- | --- | --- | --- | --- |
| Fixed | Constant | 0.1 | Low | 1000 | 0 | NA | Concave | Exponential |
| Fixed | Constant | 0.2 | Low | 1000 | 0 | NA | Concave | Exponential |
| Fixed | Constant | 0.3 | Low | 1000 | 0 | NA | Concave | Exponential |
| Fixed | Constant | 0.1 | Medium | 1000 | 1 | A | Concave | Exponential |
| Fixed | Constant | 0.1 | Medium | 1000 | 1 | B | Convex | Quadratic |
| Fixed | Constant | 0.2 | Medium | 1000 | 1 | A | Concave | Exponential |
| Fixed | Constant | 0.2 | Medium | 1000 | 1 | B | Convex | Quadratic |
| Fixed | Constant | 0.3 | Medium | 1000 | 1 | A | Concave | Exponential |
| Fixed | Constant | 0.3 | Medium | 1000 | 1 | B | Convex | Quadratic |
| Fixed | Constant | 0.1 | High | 1000 | 1 | A | Linear | Linear |
| Fixed | Constant | 0.1 | High | 1000 | 1 | B | Linear | Linear |
| Fixed | Constant | 0.2 | High | 1000 | 1 | A | Concave | Quadratic |
| Fixed | Constant | 0.2 | High | 1000 | 1 | B | Convex | Quadratic |
| Fixed | Constant | 0.3 | High | 1000 | 1 | A | Concave | Exponential |
| Fixed | Constant | 0.3 | High | 1000 | 1 | B | Convex | Quadratic |
| Fixed | Constant | 0.1 | Low | 500 | 1 | A | Linear | Linear |
| Fixed | Constant | 0.1 | Low | 500 | 1 | B | Convex | Quadratic |
| Fixed | Constant | 0.2 | Low | 500 | 1 | A | Linear | Linear |
| Fixed | Constant | 0.2 | Low | 500 | 1 | B | Convex | Quadratic |
| Fixed | Constant | 0.3 | Low | 500 | 0 | NA | Concave | Quadratic |
| Fixed | Constant | 0.1 | Medium | 500 | 1 | A | Linear | Linear |
| Fixed | Constant | 0.1 | Medium | 500 | 1 | B | Convex | Quadratic |
| Fixed | Constant | 0.2 | Medium | 500 | 1 | A | Linear | Linear |
| Fixed | Constant | 0.2 | Medium | 500 | 1 | B | Convex | Quadratic |
| Fixed | Constant | 0.3 | Medium | 500 | 1 | A | Linear | Linear |
| Fixed | Constant | 0.3 | Medium | 500 | 1 | B | Convex | Quadratic |
| Fixed | Constant | 0.1 | High | 500 | 0 | NA | Convex | Quadratic |
| Fixed | Constant | 0.2 | High | 500 | 1 | A | Linear | Linear |
| Fixed | Constant | 0.2 | High | 500 | 1 | B | Convex | Quadratic |
| Fixed | Constant | 0.3 | High | 500 | 1 | A | Convex | Linear |
| Fixed | Increasing | 0.1 | Low | 1000 | 0 | NA | Convex | Quadratic |
| Fixed | Increasing | 0.2 | Low | 1000 | 0 | NA | Convex | Quadratic |
| Fixed | Increasing | 0.3 | Low | 1000 | 0 | NA | Convex | Quadratic |
| Fixed | Increasing | 0.1 | Medium | 1000 | 0 | NA | Convex | Quadratic |
| Fixed | Increasing | 0.2 | Medium | 1000 | 0 | NA | Convex | Quadratic |
| Fixed | Increasing | 0.3 | Medium | 1000 | 0 | NA | Convex | Quadratic |
| Fixed | Increasing | 0.1 | High | 1000 | 0 | NA | Convex | Quadratic |
| Fixed | Increasing | 0.2 | High | 1000 | 0 | NA | Convex | Quadratic |
| Fixed | Increasing | 0.3 | High | 1000 | 0 | NA | Convex | Quadratic |
| Fixed | Increasing | 0.1 | Low | 500 | 0 | NA | Convex, hump | Quadratic |
| Fixed | Increasing | 0.2 | Low | 500 | 0 | NA | Convex, hump | Quadratic |
| Fixed | Increasing | 0.3 | Low | 500 | 0 | NA | Convex, hump | Quadratic |
| Fixed | Increasing | 0.1 | Medium | 500 | 0 | NA | Convex, hump | Quadratic |
| Fixed | Increasing | 0.2 | Medium | 500 | 0 | NA | Convex, hump | Quadratic |
| Fixed | Increasing | 0.3 | Medium | 500 | 0 | NA | Convex, hump | Quadratic |
| Fixed | Increasing | 0.1 | High | 500 | 0 | NA | Convex, hump | Quadratic |
| Fixed | Increasing | 0.2 | High | 500 | 0 | NA | Convex, hump | Quadratic |
| Fixed | Increasing | 0.3 | High | 500 | 0 | NA | Convex, hump | Quadratic |
| Fixed | Deceasing | 0.1 | Low | 1000 | 1 | A | Concave | Quadratic |
| Fixed | Deceasing | 0.1 | Low | 1000 | 1 | B | Linear | Linear |
| Fixed | Deceasing | 0.2 | Low | 1000 | 1 | A | Concave | Exponential |
| Fixed | Deceasing | 0.2 | Low | 1000 | 1 | B | Convex | Quadratic |
| Fixed | Deceasing | 0.3 | Low | 1000 | 0 | NA | Concave | Exponential |
| Fixed | Deceasing | 0.1 | Medium | 1000 | 1 | A | Concave | Exponential |
| Fixed | Deceasing | 0.1 | Medium | 1000 | 1 | B | Convex | Quadratic |
| Fixed | Deceasing | 0.2 | Medium | 1000 | 1 | A | Concave | Exponential |
| Fixed | Deceasing | 0.2 | Medium | 1000 | 1 | B | Convex | Quadratic |
| Fixed | Deceasing | 0.3 | Medium | 1000 | 1 | A | Concave | Quadratic |
| Fixed | Deceasing | 0.1 | High | 1000 | 0 | A | Concave | Exponential |
| Fixed | Deceasing | 0.2 | High | 1000 | 0 | A | Concave | Quadratic |
| Fixed | Deceasing | 0.3 | High | 1000 | 1 | A | Concave | Quadratic |
| Fixed | Deceasing | 0.3 | High | 1000 | 1 | B | Convex, up to K | Quadratic |
| Fixed | Decreasing | 0.1 | Low | 500 | 1 | A | Concave | Quadratic |
| Fixed | Decreasing | 0.1 | Low | 500 | 1 | B | Convex | Linear |
| Fixed | Deceasing | 0.2 | Low | 500 | 1 | A | Linear | Linear |
| Fixed | Deceasing | 0.2 | Low | 500 | 1 | B | Convex | Quadratic |
| Fixed | Deceasing | 0.3 | Low | 500 | 0 | NA | Concave | Quadratic |
| Fixed | Deceasing | 0.1 | Medium | 500 | 1 | A | Linear | Linear |
| Fixed | Deceasing | 0.1 | Medium | 500 | 1 | B | Convex | Quadratic |
| Fixed | Deceasing | 0.2 | Medium | 500 | 1 | A | Concave | Quadratic |
| Fixed | Deceasing | 0.2 | Medium | 500 | 1 | B | Convex | Quadratic |
| Fixed | Deceasing | 0.3 | Medium | 500 | 1 | A | Concave | Quadratic |
| Fixed | Deceasing | 0.3 | Medium | 500 | 1 | B | Convex | Quadratic |
| Fixed | Deceasing | 0.1 | High | 500 | 0 | A | Concave | Exponential |
| Fixed | Deceasing | 0.2 | High | 500 | 1 | A | Concave | Quadratic |
| Fixed | Deceasing | 0.3 | High | 500 | 1 | A | Concave | Quadratic |
| Fixed | Deceasing | 0.3 | High | 500 | 1 | B | Convex | Quadratic |

**Table S4.** Chi-square results from two-sample test for equality of proportions between the best-fit results in null population models and those in low, constant, proportional pressure scenarios (*P1*). Significant differences at α= 0.05 are indicated by a star.

|  |  | **Exponential curve diagnosis** | | | **Concave curve diagnosis** | | |
| --- | --- | --- | --- | --- | --- | --- | --- |
| **Degradation type** | **Specific degradation** | **χ^2^** | ***d.f.*** | **p-value** | **χ^2^** | ***d.f.*** | **p-value** |
| Years either side of pressure | 25 (None) | 298.52 | 1 | <0.001* | 92.85 | 1 | <0.001* |
| Years either side of pressure | 20 | 351.24 | 1 | <0.001* | 99.66 | 1 | <0.001* |
| Years either side of pressure | 15 | 374.59 | 1 | <0.001* | 98.28 | 1 | <0.001* |
| Years either side of pressure | 10 | 463.36 | 1 | <0.001* | 104.76 | 1 | <0.001* |
| Years either side of pressure | 5 | 359.63 | 1 | <0.001* | 150.8 | 1 | <0.001* |
| Years after pressure | 20 | 338.22 | 1 | <0.001* | 92.85 | 1 | <0.001* |
| Years after pressure | 15 | 378.34 | 1 | <0.001* | 89.28 | 1 | <0.001* |
| Years after pressure | 10 | 414.08 | 1 | <0.001* | 90.17 | 1 | <0.001* |
| Years after pressure | 5 | 345.65 | 1 | <0.001* | 73.91 | 1 | <0.001* |
| Years before pressure | 20 | 311.39 | 1 | <0.001* | 103.36 | 1 | <0.001* |
| Years before pressure | 15 | 327.09 | 1 | <0.001* | 104.29 | 1 | <0.001* |
| Years before pressure | 10 | 347.51 | 1 | <0.001* | 116.7 | 1 | <0.001* |
| Years before pressure | 5 | 358.7 | 1 | <0.001* | 139.79 | 1 | <0.001* |
| Years before pressure | 2 | 0.76 | 1 | 0.383 | 4.86 | 1 | 0.027* |
| Years between monitoring | 1 | 338.22 | 1 | <0.001* | 95.55 | 1 | <0.001* |
| Years between monitoring | 2 | 284.79 | 1 | <0.001* | 95.1 | 1 | <0.001* |
| Years between monitoring | 3 | 272.94 | 1 | <0.001* | 93.29 | 1 | <0.001* |
| Years between monitoring | 5 | 160.02 | 1 | <0.001* | 20.05 | 1 | <0.001* |
| Years between monitoring | 8 | 26.67 | 1 | <0.001* | 0.36 | 1 | 0.551 |
| Increase in observation error | 1 | 139.9 | 1 | <0.001* | 54.17 | 1 | <0.001* |
| Increase in observation error | 1.5 | 77.7 | 1 | <0.001* | 52.44 | 1 | <0.001* |
| Increase in observation error | 2 | 59.83 | 1 | <0.001* | 46.8 | 1 | <0.001* |
| Increase in observation error | 2.5 | 10.92 | 1 | 0.001* | 28.56 | 1 | <0.001* |

**Table S5.** Chi-square results from two-sample test for equality of proportions between best-fit frequencies for each degradation scenario in populations with low, constant, proportional pressure (*P1*). Significant differences at α= 0.05 are indicated by a star.

|  |  | **Linear vs. quadratic fit** | | | **Linear vs. exponential fit** | | | **Quadratic vs. exponential fit** | | |
| --- | --- | --- | --- | --- | --- | --- | --- | --- | --- | --- |
| **Degradation type** | **Specific degradation** | **χ^2^** | ***d.f.*** | **p-value** | **χ^2^** | ***d.f.*** | **p-value** | **χ^2^** | ***d.f.*** | **p-value** |
| Years either side of pressure | 25 (None) | 259.32 | 1 | <0.001* | 329.29 | 1 | <0.001* | 6.04 | 1 | 0.014* |
| Years either side of pressure | 20 | 215.60 | 1 | <0.001* | 389.83 | 1 | <0.001* | 37.04 | 1 | <0.001* |
| Years either side of pressure | 15 | 221.23 | 1 | <0.001* | 436.57 | 1 | <0.001* | 54.11 | 1 | <0.001* |
| Years either side of pressure | 10 | 65.50 | 1 | <0.001* | 455.39 | 1 | <0.001* | 218.11 | 1 | <0.001* |
| Years either side of pressure | 5 | 427.01 | 1 | <0.001* | 566.01 | 1 | <0.001* | 19.34 | 1 | <0.001* |
| Years after pressure | 20 | 206.25 | 1 | <0.001* | 359.33 | 1 | <0.001* | 29.76 | 1 | <0.001* |
| Years after pressure | 15 | 253.84 | 1 | <0.001* | 469.23 | 1 | <0.001* | 51.55 | 1 | <0.001* |
| Years after pressure | 10 | 101.94 | 1 | <0.001* | 404.25 | 1 | <0.001* | 129.14 | 1 | <0.001* |
| Years after pressure | 5 | 253.13 | 1 | <0.001* | 408.87 | 1 | <0.001* | 28.12 | 1 | <0.001* |
| Years before pressure | 20 | 168.17 | 1 | <0.001* | 283.64 | 1 | <0.001* | 19.63 | 1 | <0.001* |
| Years before pressure | 15 | 300.16 | 1 | <0.001* | 410.59 | 1 | <0.001* | 13.62 | 1 | <0.001* |
| Years before pressure | 10 | 242.31 | 1 | <0.001* | 403.92 | 1 | <0.001* | 30.68 | 1 | <0.001* |
| Years before pressure | 5 | 202.81 | 1 | <0.001* | 393.08 | 1 | <0.001* | 44.65 | 1 | <0.001* |
| Years before pressure | 2 | 286.15 | 1 | <0.001* | 29.80 | 1 | <0.001* | 447.90 | 1 | <0.001* |
| Years between monitoring | 1 | 239.87 | 1 | <0.001* | 385.29 | 1 | <0.001* | 25.28 | 1 | <0.001* |
| Years between monitoring | 2 | 247.53 | 1 | <0.001* | 297.00 | 1 | <0.001* | 3.16 | 1 | 0.075 |
| Years between monitoring | 3 | 213.76 | 1 | <0.001* | 253.33 | 1 | <0.001* | 2.24 | 1 | 0.135 |
| Years between monitoring | 5 | 78.91 | 1 | <0.001* | 32.06 | 1 | <0.001* | 10.99 | 1 | <0.001* |
| Years between monitoring | 8 | 195.70 | 1 | <0.001* | 6.30 | 1 | 0.012* | 261.41 | 1 | <0.001* |
| Increase in observation error | 1 | 189.47 | 1 | <0.001* | 56.60 | 1 | <0.001* | 44.04 | 1 | <0.001* |
| Increase in observation error | 1.5 | 333.33 | 1 | <0.001* | 38.38 | 1 | <0.001* | 167.04 | 1 | <0.001* |
| Increase in observation error | 2 | 391.23 | 1 | <0.001* | 37.68 | 1 | <0.001* | 216.75 | 1 | <0.001* |
| Increase in observation error | 2.5 | 413.28 | 1 | <0.001* | 0.10 | 1 | 0.757 | 403.95 | 1 | <0.001* |

**Table S6.** Chi-square results from two-sample test for equality of proportions between the concavity frequencies diagnosed for each degradation scenario in populations with low, constant, proportional pressure (*P1*). Significant differences at α= 0.05 are indicated by a star.

|  |  | **Concave vs. Convex fit** | | |
| --- | --- | --- | --- | --- |
| **Degradation type** | **Specific degradation** | **χ^2^** | ***d.f.*** | **p-value** |
| Years either side of pressure | 25 (None) | 293.76 | 1 | <0.001* |
| Years either side of pressure | 20 | 630.44 | 1 | <0.001* |
| Years either side of pressure | 15 | 620.94 | 1 | <0.001* |
| Years either side of pressure | 10 | 665.86 | 1 | <0.001* |
| Years either side of pressure | 5 | 1000.00 | 1 | <0.001* |
| Years after pressure | 20 | 583.70 | 1 | <0.001* |
| Years after pressure | 15 | 559.50 | 1 | <0.001* |
| Years after pressure | 10 | 565.50 | 1 | <0.001* |
| Years after pressure | 5 | 456.98 | 1 | <0.001* |
| Years before pressure | 20 | 656.10 | 1 | <0.001* |
| Years before pressure | 15 | 662.60 | 1 | <0.001* |
| Years before pressure | 10 | 749.96 | 1 | <0.001* |
| Years before pressure | 5 | 917.76 | 1 | <0.001* |
| Years before pressure | 1 | 602.18 | 1 | <0.001* |
| Years between monitoring | 2 | 599.08 | 1 | <0.001* |
| Years between monitoring | 3 | 586.76 | 1 | <0.001* |
| Years between monitoring | 5 | 123.90 | 1 | <0.001* |
| Years between monitoring | 8 | 0.02 | 1 | 0.899 |
| Years between monitoring | 1 | 330.16 | 1 | <0.001* |
| Increase in observation error | 1.5 | 319.27 | 1 | <0.001* |
| Increase in observation error | 2 | 284.23 | 1 | <0.001* |
| Increase in observation error | 2.5 | 173.82 | 1 | <0.001* |

**Table S7**. Chi-square results from two-sample test for equality of proportions between the best-fit results in null population models and those in increasing fixed pressure scenarios (*F3*). Significant differences at α= 0.05 are indicated by a star.

|  |  | **Quadratic curve diagnosis** | | | **Convex curve diagnosis** | | |
| --- | --- | --- | --- | --- | --- | --- | --- |
| **Degradation type** | **Specific degradation** | **χ^2^** | ***d.f.*** | **p-value** | **χ^2^** | ***d.f.*** | **p-value** |
| Years either side of pressure | 25 (None) | 59.06 | 1 | <0.001* | 177.81 | 1 | <0.001* |
| Years either side of pressure | 20 | 20.09 | 1 | <0.001* | 150.91 | 1 | <0.001* |
| Years either side of pressure | 15 | 22.31 | 1 | <0.001* | 146.57 | 1 | <0.001* |
| Years either side of pressure | 10 | 23.46 | 1 | <0.001* | 152.55 | 1 | <0.001* |
| Years either side of pressure | 5 | 49.79 | 1 | <0.001* | 144.41 | 1 | <0.001* |
| Years after pressure | 20 | 20.09 | 1 | <0.001* | 140.13 | 1 | <0.001* |
| Years after pressure | 15 | 16.55 | 1 | <0.001* | 134.84 | 1 | <0.001* |
| Years after pressure | 10 | 16.36 | 1 | <0.001* | 148.74 | 1 | <0.001* |
| Years after pressure | 5 | 45.1 | 1 | <0.001* | 127.03 | 1 | <0.001* |
| Years before pressure | 20 | 27.06 | 1 | <0.001* | 162.51 | 1 | <0.001* |
| Years before pressure | 15 | 30.35 | 1 | <0.001* | 153.1 | 1 | <0.001* |
| Years before pressure | 10 | 27.06 | 1 | <0.001* | 152.55 | 1 | <0.001* |
| Years before pressure | 5 | 29.32 | 1 | <0.001* | 164.75 | 1 | <0.001* |
| Years before pressure | 2 | 27.06 | 1 | <0.001* | 152.01 | 1 | <0.001* |
| Years between monitoring | 1 | 23.23 | 1 | <0.001* | 138.54 | 1 | <0.001* |
| Years between monitoring | 2 | 22.31 | 1 | <0.001* | 147.11 | 1 | <0.001* |
| Years between monitoring | 3 | 33 | 1 | <0.001* | 149.28 | 1 | <0.001* |
| Years between monitoring | 5 | 33 | 1 | <0.001* | 153.1 | 1 | <0.001* |
| Years between monitoring | 8 | 45.71 | 1 | <0.001* | 179.54 | 1 | <0.001* |
| Increase in observation error | 1 | 50.11 | 1 | <0.001* | 139.07 | 1 | <0.001* |
| Increase in observation error | 1.5 | 48.21 | 1 | <0.001* | 141.2 | 1 | <0.001* |
| Increase in observation error | 2 | 50.76 | 1 | <0.001* | 122.42 | 1 | <0.001* |
| Increase in observation error | 2.5 | 51.73 | 1 | <0.001* | 123.44 | 1 | <0.001* |

**Table S8.** Chi-square results from two-sample test for equality of proportions between best-fit frequencies for each degradation scenario in populations with increasing, fixed pressure (*F3*). Significant differences at α= 0.05 are indicated by a star.

|  |  | **Linear vs. quadratic fit** | | | **Linear vs. exponential fit** | | | **Quadratic vs. exponential fit** | | |
| --- | --- | --- | --- | --- | --- | --- | --- | --- | --- | --- |
| **Degradation type** | **Specific degradation** | **χ^2^** | ***d.f.*** | **p-value** | **χ^2^** | ***d.f.*** | **p-value** | **χ^2^** | ***d.f.*** | **p-value** |
| Years either side of pressure | 25 (None) | 933.16 | 1 | <0.001* | 17.00 | 1 | <0.001* | 983.00 | 1 | <0.001* |
| Years either side of pressure | 20 | 476.10 | 1 | <0.001* | 155.00 | 1 | <0.001* | 845.00 | 1 | <0.001* |
| Years either side of pressure | 15 | 506.03 | 1 | <0.001* | 141.03 | 1 | <0.001* | 852.00 | 1 | <0.001* |
| Years either side of pressure | 10 | 524.30 | 1 | <0.001* | 128.26 | 1 | <0.001* | 851.04 | 1 | <0.001* |
| Years either side of pressure | 5 | 836.08 | 1 | <0.001* | 2.00 | 1 | 0.157 | 868.65 | 1 | <0.001* |
| Years after pressure | 20 | 477.96 | 1 | <0.001* | 151.03 | 1 | <0.001* | 842.00 | 1 | <0.001* |
| Years after pressure | 15 | 435.59 | 1 | <0.001* | 160.21 | 1 | <0.001* | 819.04 | 1 | <0.001* |
| Years after pressure | 10 | 434.70 | 1 | <0.001* | 157.37 | 1 | <0.001* | 815.08 | 1 | <0.001* |
| Years after pressure | 5 | 802.71 | 1 | <0.001* | 24.07 | 1 | <0.001* | 907.51 | 1 | <0.001* |
| Years before pressure | 20 | 562.50 | 1 | <0.001* | 125.00 | 1 | <0.001* | 875.00 | 1 | <0.001* |
| Years before pressure | 15 | 604.33 | 1 | <0.001* | 108.04 | 1 | <0.001* | 885.00 | 1 | <0.001* |
| Years before pressure | 10 | 562.50 | 1 | <0.001* | 125.00 | 1 | <0.001* | 875.00 | 1 | <0.001* |
| Years before pressure | 5 | 589.82 | 1 | <0.001* | 116.00 | 1 | <0.001* | 884.00 | 1 | <0.001* |
| Years before pressure | 2 | 564.57 | 1 | <0.001* | 121.03 | 1 | <0.001* | 872.00 | 1 | <0.001* |
| Years between monitoring | 1 | 834.40 | 1 | <0.001* | 40.09 | 1 | <0.001* | 953.00 | 1 | <0.001* |
| Years between monitoring | 2 | 817.86 | 1 | <0.001* | 38.72 | 1 | <0.001* | 941.04 | 1 | <0.001* |
| Years between monitoring | 3 | 852.50 | 1 | <0.001* | 24.38 | 1 | <0.001* | 943.10 | 1 | <0.001* |
| Years between monitoring | 5 | 852.78 | 1 | <0.001* | 35.10 | 1 | <0.001* | 958.00 | 1 | <0.001* |
| Years between monitoring | 8 | 519.44 | 1 | <0.001* | 133.11 | 1 | <0.001* | 853.02 | 1 | <0.001* |
| Increase in observation error | 1 | 507.96 | 1 | <0.001* | 137.11 | 1 | <0.001* | 849.02 | 1 | <0.001* |
| Increase in observation error | 1.5 | 635.84 | 1 | <0.001* | 98.04 | 1 | <0.001* | 895.00 | 1 | <0.001* |
| Increase in observation error | 2 | 640.32 | 1 | <0.001* | 90.35 | 1 | <0.001* | 889.04 | 1 | <0.001* |
| Increase in observation error | 2.5 | 781.46 | 1 | <0.001* | 58.00 | 1 | <0.001* | 942.00 | 1 | <0.001* |

**Table S9.** Chi-square results from two-sample test for equality of proportions between the concavity frequencies diagnosed for each degradation scenario in populations with increasing, fixed pressure (*F3*). Significant differences at α= 0.05 are indicated by a star.

|  |  | **Concave vs. Convex fit** | | |
| --- | --- | --- | --- | --- |
| **Degradation type** | **Specific degradation** | **χ^2^** | ***d.f.*** | **p-value** |
| Years either side of pressure | 25 (None) | 960.40 | 1 | <0.001* |
| Years either side of pressure | 20 | 781.46 | 1 | <0.001* |
| Years either side of pressure | 15 | 753.42 | 1 | <0.001* |
| Years either side of pressure | 10 | 792.10 | 1 | <0.001* |
| Years either side of pressure | 5 | 739.60 | 1 | <0.001* |
| Years after pressure | 20 | 712.34 | 1 | <0.001* |
| Years after pressure | 15 | 678.98 | 1 | <0.001* |
| Years after pressure | 10 | 767.38 | 1 | <0.001* |
| Years after pressure | 5 | 630.44 | 1 | <0.001* |
| Years before pressure | 20 | 857.48 | 1 | <0.001* |
| Years before pressure | 15 | 795.66 | 1 | <0.001* |
| Years before pressure | 10 | 792.10 | 1 | <0.001* |
| Years before pressure | 5 | 872.36 | 1 | <0.001* |
| Years before pressure | 2 | 788.54 | 1 | <0.001* |
| Years between monitoring | 1 | 705.60 | 1 | <0.001* |
| Years between monitoring | 2 | 719.10 | 1 | <0.001* |
| Years between monitoring | 3 | 602.18 | 1 | <0.001* |
| Years between monitoring | 5 | 608.40 | 1 | <0.001* |
| Years between monitoring | 8 | 702.24 | 1 | <0.001* |
| Increase in observation error | 1 | 756.90 | 1 | <0.001* |
| Increase in observation error | 1.5 | 770.88 | 1 | <0.001* |
| Increase in observation error | 2 | 795.66 | 1 | <0.001* |
| Increase in observation error | 2.5 | 972.20 | 1 | <0.001* |
